# Volatile organic compound analysis of malignant pleural mesothelioma chorioallantoic membrane xenografts

**DOI:** 10.1088/1752-7163/ad7166

**Published:** 2024-09-11

**Authors:** Liam D Little, Sarah E Barnett, Theo Issitt, Sam Bonsall, Vikki A Carolan, Elizabeth Allen, Laura M Cole, Neil A Cross, Judy M Coulson, Sarah L Haywood-Small

**Affiliations:** 1 Biomolecular Sciences Research Centre, Department of Biosciences and Chemistry, Faculty of Health and Wellbeing, Sheffield Hallam University, Sheffield S1 1WB, United Kingdom; 2 Egg Facility, Liverpool Shared Research Facilities, Technology Infrastructure and Environment Directorate, University of Liverpool, Liverpool, United Kingdom; 3 Department of Molecular and Clinical Cancer Medicine, Institute of Systems Molecular and Integrative Biology, University of Liverpool, Liverpool, United Kingdom

**Keywords:** mesothelioma, volatile organic compounds, CAM model, xenograft, biomarkers, cancer biomarkers

## Abstract

Malignant pleural mesothelioma (MPM) is an aggressive cancer associated with asbestos exposure. MPM is often diagnosed late, at a point where limited treatment options are available, but early intervention could improve the chances of successful treatment for MPM patients. Biomarkers to detect MPM in at-risk individuals are needed to implement early diagnosis technologies. Volatile organic compounds (VOCs) have previously shown diagnostic potential as biomarkers when analysed in MPM patient breath. In this study, chorioallantoic membrane (CAM) xenografts of MPM cell lines were used as models of MPM tumour development for VOC biomarker discovery with the aim of generating targets for investigation in breath, biopsies or other complex matrices. VOC headspace analysis of biphasic or epithelioid MPM CAM xenografts was performed using solid-phase microextraction and gas chromatography-mass spectrometry. We successfully demonstrated the capture, analysis and separation of VOC signatures from CAM xenografts and controls. A panel of VOCs was identified that showed discrimination between MPM xenografts generated from biphasic and epithelioid cells and CAM controls. This is the first application of the CAM xenograft model for the discovery of VOC biomarkers associated with MPM histological subtypes. These findings support the potential utility of non-invasive VOC profiling from breath or headspace analysis of tissues for detection and monitoring of MPM.

## 1. Introduction

Malignant pleural mesothelioma (MPM) is an aggressive malignancy with a well-established link between previous asbestos exposure, which is commonly occupational, and MPM development [1]. Despite regulations and controls regarding asbestos, rates of MPM deaths have yet to decline and initial exposure to asbestos fibres initiates a prolonged latency period of up to 50 years, before MPM diagnosis [2]. Symptoms are non-specific (breathlessness, chest pains, pleural effusions) but usually represent MPM at a late stage where treatment options are limited [3]. Currently, diagnosis involves radiography and computed tomography (CT) scans, but MPM must be confirmed through histological examination following an invasive biopsy procedure [3]. Therefore, alternate methods of diagnosis are necessary to identify MPM at an earlier stage and improve patient outcomes.

Volatile organic compounds (VOCs) are chemical compounds present in exhaled breath which show great potential for disease diagnosis and monitoring [4, 5]. Measuring VOCs can reveal valuable information directly related to cellular metabolism and metabolic dysregulation, with changes in the patterns and levels of VOCs present in breath linked to several diseases [6]. Breath analysis has therefore seen widespread research within cancer diagnostics, however breath-based biomarkers have not yet been adopted into routine clinical practice [7]. In MPM, analysis of VOC profiles has been used to distinguish MPM patients from other clinical groups, such as patients with other asbestos-related diseases or healthy controls [8–10]. Recently, measurement of VOCs with an electronic nose produced promising results for determining MPM patient response and monitoring following immunotherapy [11]. These studies highlight the possibility of using VOC analysis to aid in the diagnosis of MPM. The culmination of this field of research would be the development of a non-invasive breath test that could sensitively, specifically, and reliably detect MPM at an earlier stage than current methods.

MPM cell lines have also been used as a model for breath analysis by detecting VOCs released into the headspace gas above two-dimensional (2D) *in vitro* cell cultures [12]. Established biphasic and epithelioid MPM cell cultures produced distinct VOC profiles and specific compounds were identified that correlated between studies [12, 13]. *In vitro* model systems facilitate functional experiments to test hypotheses that could not easily be addressed utilising patient breath samples directly, such as chemotherapeutic stress [14] and hypoxic biomarker discovery [15]. These complementary *in vitro* approaches can be used to substantiate and augment *in vivo* investigations and elucidate the characteristic volatile fingerprints of disease states. However, 2D *in vitro* cell culture cannot recapitulate aspects of a human tumour, where cells are growing in three-dimensions (3Ds) and interacting with the tumour microenvironment, including stromal cells and blood vessels.

The aim of the current study was to further develop methods and models for cell to breath translatable VOC biomarker discovery. The chick chorioallantoic membrane (CAM) is an extraembryonic membrane responsible for nutrient and gaseous exchange via the eggshell, that is distinct from the yolk sac and its vascularised membrane [16]. The CAM is easily accessible via a small opening cut in the eggshell and is an excellent substrate for generating complex 3D vascularised tumour nodules that better mimic human disease [16, 17]. This study uses CAM xenografts, a more complex model of MPM than 2D cell culture, for the discovery of biomarkers which can be targeted in more complex matrices, such as breath and tissue, from patients. For this study, three MPM cell lines derived from patients with epithelioid or biphasic disease [18] were engrafted on the CAM and VOCs were extracted from excised tumour nodules. This work demonstrates the potential of this model for defining VOC biomarkers of MPM. Furthermore, we present a panel of VOCs able to separate MPM CAM xenografts from controls and distinguish epithelioid from biphasic MPM for further investigation in patient samples.

## 2. Methods

### 2.1. Cell culture

The MESO-7 T (7 T), MESO-8 T (8 T) and MESO-12 T (12 T) mesothelioma cell lines obtained from Mesobank [18, 19] were authenticated by STR-profiling (ATCC) and shown to be mycoplasma-free. They were maintained, as previously described [18], in RPMI 1640 Glutamax (Thermo Scientific, Walham, MA, USA) supplemented with 10% fetal bovine serum (FBS), 20 ng ul^−1^ EGF (Peprotech, Altrincham, UK), 1 mg ml^−1^ hydrocortisone and 2 mg ml^−1^ heparin (Sigma-Aldrich, Mannheim, Germany) in a 5% CO_2_ humidified incubator at 37 °C. Dual-labelled (fluorescent/luminescent) mesothelioma cell lines were generated with pHIV-Luc-ZsGreen (Addgene plasmid #39196; http://n2t.net/addgene:39196) as previously described [16].

### 2.2. MPM CAM model

Fertilised Shaver Brown eggs (Medeggs Ltd, Fakenham, UK) were incubated at 37 °C and 45% humidity (embryonic day 0; E0) and windowed at E3. Prior to implantation on E7, trypsinised dual-labelled MPM cells were counted, washed in sterile PBS, and pelleted via centrifugation. Standard protocols were followed [16] with 2 × 10^6^ cells implanted per egg. On E14, viability of the established tumour nodules on the CAM were assessed by bioluminescent imaging. Briefly, 250 *µ*l of 15 mg ml^−1^ luciferin (Promega, Madison, WI, USA) was injected into the yolk sac and bioluminescent signal measured following 45 min incubation using the IVIS Spectrum *In Vivo* Imaging System (Perkin Elmer, Bucks, UK). Tumours were then observed under a Leica M165FC fluorescent stereomicroscope with 16.5:1 zoom optics, fitted with a Leica DFC425 C camera. Brightfield and fluorescent images were acquired both prior to and post dissection. Harvested tumours (12 T *n* = 7, 8 T *n* = 9, 7 T *n* = 5) were weighed, placed in 1 ml RNAlater (Thermo Fisher, Loughborough, UK) and frozen at −80 °C immediately. RNAlater is a pH 8, tissue and cell preservative suitable for freezing that was previously used successfully for this purpose [20]. Following removal of the tumour nodules, embryos were terminated on E14. Control (*n* = 5) and mock (*n* = 5) samples were collected in parallel.

Control and mock CAM samples were used as experimental controls to determine whether the CAM itself or traumatisation of the CAMprior to implanting cellscould be a potentialsource of VOCs. Control samples were taken from eggs where the CAM was left untreated from E3 to E14. Mock samples were taken from eggs where the CAM underwent the standard implant preparation procedure on E7, but no cells were added, with the site that was traumatised being excised. For both, small sections of CAM (roughly 2–4 mm) were excised at E14 for VOC extraction.

### 2.3. VOC extraction

VOCs were extracted from samples using a method previously published for the extraction of compounds from lung carcinoma samples [20]. CAM samples in RNAlater were thawed on ice prior to extraction. For each sample, 200 *µ*l of RNAlater only was transferred to a 2 ml headspace vial with PTFE/silicon caps to serve as a background control. The remaining RNAlater plus samples were transferred to a second 2 ml glass headspace vial. Sealed headspace vials were incubated at 37 °C for 60 min with continuous solid phase microextraction (SPME) throughout the incubation. For SPME, a new 50/30 *µ*m divinylbenzene/Carboxen/polydimethylsiloxane (DVB/CAR/PDMS) fibre with manual fibre assembly (Supelco, Bellefonte, PA, USA) was conditioned at 270 °C for 30 min prior to initial use and further conditioned each day before extraction for 10 min at 250 °C. For VOC extraction, the SPME fibre assembly was inserted through the septum cap of the headspace vials and the SPME fibre exposed to the headspace gas above the samples.

### 2.4. Gas chromatography-mass spectrometry

VOCs were analysed using gas chromatography mass spectrometry (GC-MS) as previously published [12]. The SPME fibre assembly was transferred to the inlet of an Agilent 7890A with a Rtx-VMS capillary column (30 m × 0.25 mm × 1.4 *µ*m; Restek, Bellefonte, PN, United States) and MS-5975 C triple axis detector. The GC-MS inlet temperature was set to 250 °C. The oven temperature programming was set to: 35 °C for 5 min, ramped to 140 °C at 4 °C min^−1^ and held for 5 min, ramped again to 240 °C at 20 °C min^−1^ and held for 4 min. The total analysis time was 45.25 min. The MS transfer line was set to 260 °C and analysis was performed in full scan mode with a range of 35–300 a.m.u. The SPME fibre assembly was manually injected into the GC-MS inlet and the fibre exposed for the first 10 min of the oven temperature program. After this 10 min exposure the SPME fibre was ready to be used again for VOC extraction.

## 3. Statistical analysis

### 3.1. Data pre-processing

GC-MS data files were exported for alignment and processing from Masshunter software (Agilent Technologies, Cheadle, UK) and similar methods followed for processing untargeted GC-MS data as previously reported [21]. Data were pre-processed through deconvolution, alignment and integration using the *eRah* package for R (v4.2.1) [22] using open format CDF files exported from Masshunter. This generated a matrix of aligned features against feature ID for further processing. The alignment matrix was uploaded to metaboanalyst where missing data were imputed with one-fifth the lowest recorded value for each feature. Data were then normalised to SUM, log transformed and scaled by mean-centring and dividing by the standard deviation of each variable. A data matrix of identified, aligned features listed by feature ID was produced with normalised peak intensity recorded for each feature.

7 T, 8 T and 12 T cell line groups and Mock/Control groups were compared to their respective RNAlater background group and Benjamini–Hochberg false discovery rate (FDR) [23] applied to identify variables which were significantly different from background groups, with a *p* value of less than 0.05. 94 significantly altered features specific to samples containing tissue were identified from a total of 1489 and used for further analysis.

Univariate and multivariate analysis was performed independently to identify differences between groups. Univariate analysis was performed to identify differences between identified compounds between groups in the form of a series of T-tests with FDR. Alongside univariate analysis, data were re-uploaded to Metaboanalyst for multivariate analysis using unsupervised principal component analysis (PCA) and supervised partial least-squares discrimination analysis (PLS-DA). PLS-DA was tuned using five-fold cross validation. Top discriminant features were identified with either a *p* < 0.05 by univariate analysis or top variable importance of projection (VIP) score. Identified features were checked against original alignment and true signals were given tentative compound identification through *eRah,* using Massbank of North America (MoNA; https://mona.fiehnlab.ucdavis.edu/) GC-MS library if confidence was above 90%. These identifications were then cross referenced against the original GC-MS data files using NIST 11 and Masshunter Qualitative Analysis software (Agilent). To support this method of feature identification, random forest classifications were also applied to the data in Metaboanalyst using 1000 trees, 7 predictors and random settings. Mean decrease accuracy scores for the top 15 features are shown in figure S8 against retention time of compounds, these scores are also presented in table 1.

**Table 1.** VOCs identified as most significantly different (*p* < 0.005) across 7 T, 8 T, 12 T, Mock and Control groups with comparison to their respective RNAlater background group. Average retention time (AVG RT), variance important projection (VIP) from PLS-DA, mean decrease accuracy scores from random forest classification and tentative IDs above 95% against the MassBank of North America (MoNA) GC-MS library.

AVG RT	VIP score	Mean decrease accuracy	Tentative identification	CAS	Calculated retention index
2.91	1.14	0.007 13	Oxalic acid	144-62-7	NA
3.59	1.07	0.004 91	Isopropanol	67-63-0	NA
3.70	1.61	0.016 28	Acetone	67-64-1	NA
3.80	1.56	0.020 58	Butane	106-97-8	NA
4.26	1.56	0.020 57	2-methyl-2-propanol	553-90-2	NA
4.35	1.39	0.022 81	Dimethyl Oxalate	75-65-0	504.33
7.11	1.78	0.011 17	2-methyl propanal	78-84-2	615.57
7.23	1.27	0.005 13	2-butanone	78-93-3	623.05
9.30	2.06	0.000 37	3-methyl cyclohexane	591-24-2	735.67
10.80	2.21	0.006 26	2-ethyl hexanol	104-76-7	802.97
11.83	2.08	0.008 34	2,3-pentanedione	600-14-6	843.3
16.23	0.35	0.016 13	Benzaldehyde	100-52-7	984.75
16.25	0.31	0.017 71	2-methoxyethanol	109-86-4	985.3
16.30	1.65	0.002 73	Hexanal	66-25-1	986.67
16.62	2.05	0.007 35	2-Aminopyridine	504-29-0	995.37
18.71	1.15	0.005 49	2,2-dimethyl-propanoic acid	75-98-9	1048.35
26.19	1.11	0.001 94	Diethylene glycol dipivalate	24 405-27-2	1198.79
37.89	1.28	0.004 14	Pentanoic acid	109-52-4	1363.98
42.28	1.74	0.004 36	Pentacosane	629-99-2	1413.02

Significantly altered identified compounds were re-uploaded to Metaboanalyst to generate receiver operating characteristics (ROCs) curves: comparisons were made between combined MPM groups (7 T, 8 T, 12 T) to combined control groups (Mock/Control) and between the 7 T group (Biphasic MPM) to 8 T and 12 T groups combined (Epithelioid MPM). Multivariate ROC curves were generated using PLS-DA classification and feature ranking in Metaboanalyst 6 using a combination of the 2–19 variables (listed in table 1) in order of their classified importance. Heat maps were also generated to visualise VOC peak intensity (log_10_) in control, mock, 7 T, 8 T and 12 T groups using Rstudio and *complexheatmap* package. Graphpad Prism (GraphPad Software, version 10, San Diego, CA, USA) was used to generate graphs of peak intensity (log10), from processed data for identified compounds.

## 4. Results

### 4.1. MPM CAM xenografts

**Figure 1.**
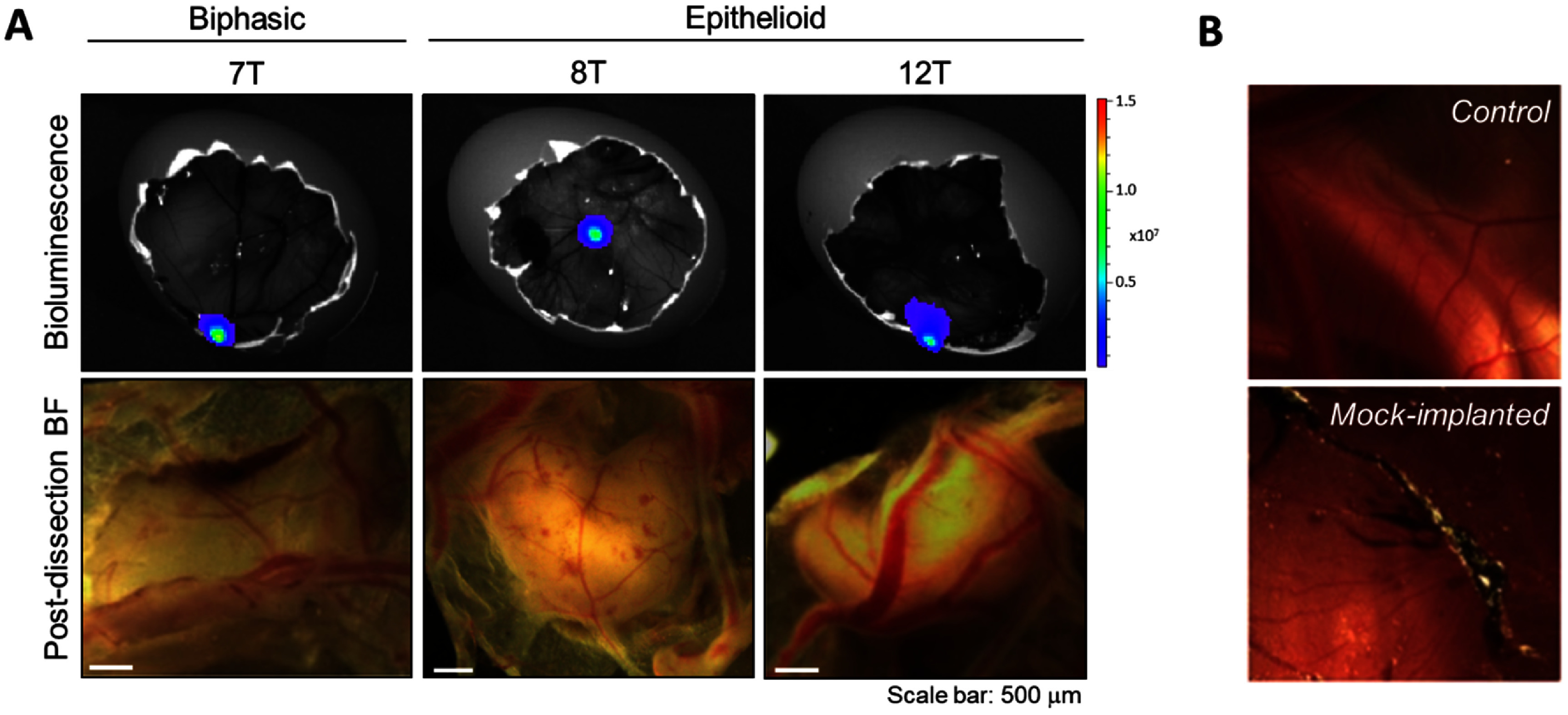
Mesothelioma xenografts generated on the CAM. (A) Representative tumour nodules for each mesothelioma cell line evaluated. Bioluminescent signal (top) and corresponding brightfield (BF) image taken post dissection (bottom). Bioluminescence signal is shown as total flux (radiance: p s^−1^ cm^−2^ sr^−1^). Colour scale: min = 3.79 × 10^5^, max = 1.151 × 10^7^. Scale bar = 500 *µ*m. (B) Representative images of CAM controls acquired prior to dissection.

Mesothelioma cell lines 7 T, 8 T and 12 T formed viable, vascularised xenografts when implanted on the CAM (figure 1(A)). All three cell lines had been modified to stably express a luciferase reporter, allowing *in vivo* visualisation and assessment of viability via bioluminescent imaging (figure 1(A)). Tumour nodule weights for each cell line were 7.0 ± 2.7 mg (MESO-7 T, *n* = 4), 13.0 ± 4.8 mg (MESO-8 T, *n* = 9) and 17.3 ± 6.7 mg (MESO-12 T, *n* = 7), respectively (supplemental figure 3(A)). To account for any background VOC signal coming from the CAM alone, or from an inflammatory response due to damage of the CAM during implantation, additional untreated control and mock samples were acquired for analysis (figure 1(B)).

### 4.2. VOCs significantly associated with MPM CAM xenografts

**Figure 2.**
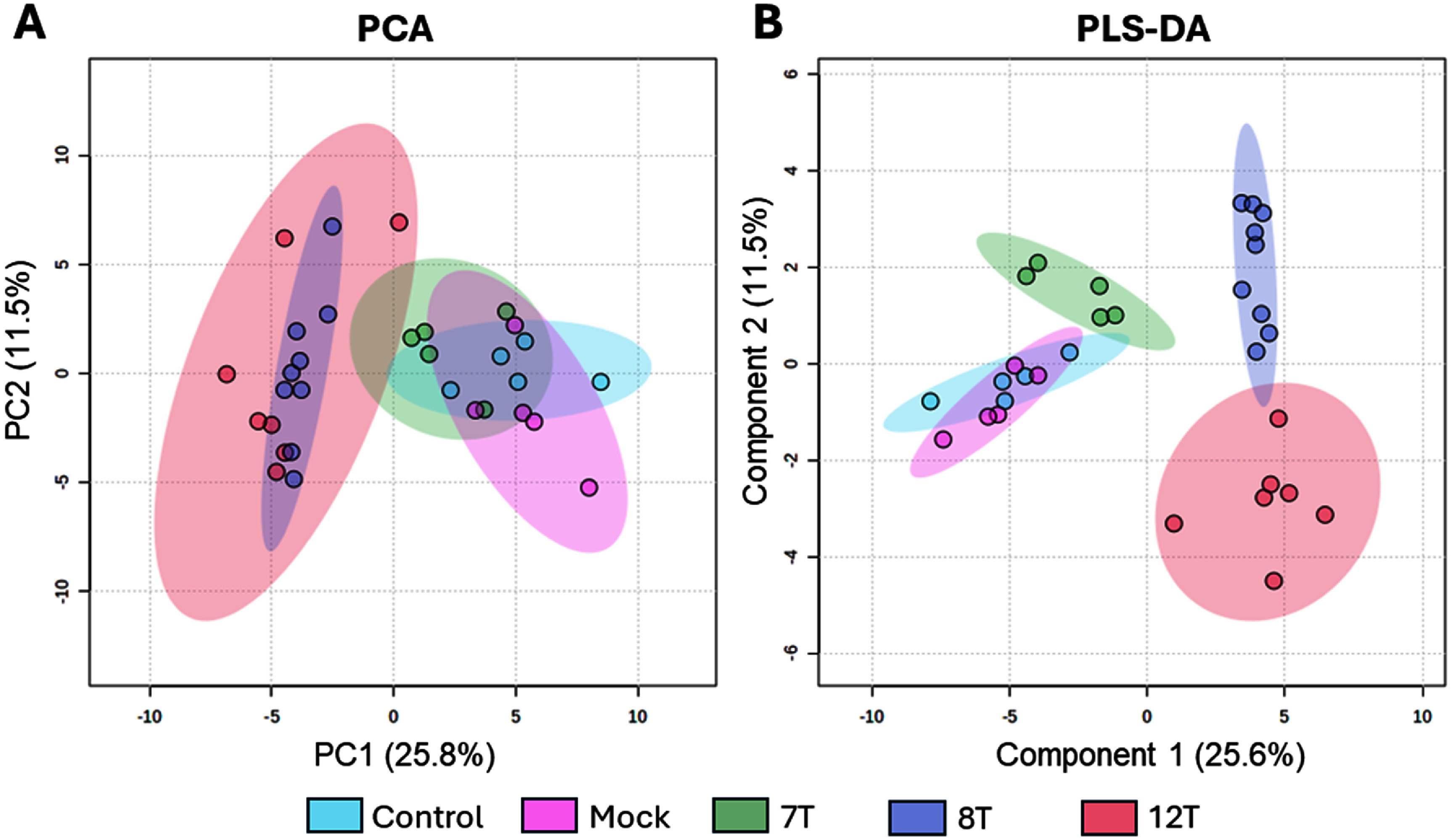
PCA (A) and PLS-DA (B) score plots of VOCs from control CAM, mock CAM and CAM xenografts of the mesothelioma cell lines 7 T, 8 T and 12 T. Each point represents the VOC profile from a single sample after normalisation and background filtering.

In order to identify VOCs originating from the biological samples, 7 T, 8 T, 12 T, Mock and Control CAM groups were compared to their respective RNAlater background groups. Batch *t*-tests were performed in Metaboanalyst comparing peak areas to identify significantly altered compounds in the CAM groups compared to their RNAlater background controls. Across all sample groups, 94 features were identified as significantly altered from respective controls. These were investigated to determine which features were most descriptive of groups using unsupervised multivariate PCA (figure 2(A)). Clear separation of the epithelioid 8 T and 12 T CAM xenografts from all other groups was observed in PCA along PC1, however these two groups were inseparable from each other. The biphasic 7 T CAM xenografts were not separable from control or mock groups via PCA (figure 2(A)). Taken together, the PCA results reveal explained variance for 8 T and 12 T along PC1 against all other groups but little other separation, considering PC3 vs PC1 (figure S1) did not explain this further. As there was little separation of experimental groups by unsupervised PCA analysis, supervised PLS-DA was used to explore features which drive separation of experimental groups (figure 2(B)).

Using supervised PLS-DA analysis, clear experimental group separation was observed for 7 T, 8 T and 12 T. However, the two control groups, untreated CAM (control) and mock-implanted CAM, remained inseparable suggesting that there was no contribution to the VOC profile from the traumatisation of the CAM used when engrafting MPM cells in the other groups. 7 T and 8 T were separable along component 2 but not component 1 from 12 T whereas 12 T and 8 T were separable from 7 T along component 1. Control and mock groups were clearly separated from 8 T and 12 T groups along component 1 and from 7 T along component 2. The 7 T cell xenografts were smaller than other cell lines and we investigate this relationship in subsequent paragraphs, however because control weights were not taken, direct comparison of weight between tumours and CAM was not possible. In both PCA and PLS-DA, control and mock CAM groups were inseparable, meaning no features were significantly different between groups (figure 2). A separate PCA (supplementary figure 1) and *T*-tests on control vs mock CAM features, extracted from the PCA loadings as significant from relative control, revealed a single feature at 16.68 min as significantly altered (*p* = 0.0473).

To determine whether the presence of luciferin, used to enable bioluminescent imaging of viable CAM xenografts might influence the VOC profile, control/mock samples from non-implanted eggs injected with luciferin were analysed. Injection of luciferin did not appear to affect the spread of the Mock/Control groups using PCA (figure S2). A single VOC with a retention time of 16.68 min was significantly changed (*p* = 0.0473) between control and mock but this compound was not a feature which was significantly altered in tumour samples. CAM tumour weight varied between samples, with 7 T xenografts the smallest (figure S3(A)), however there was no obvious effect of xenograft weight upon spread of data in the PCA between xenograft groups (figure S3(B)), but we set out to explore this further.

PCA plot of CAM xenografts only, appeared to reveal grouping, highlighted in figure S4(A). Because 7 T xenografts were smaller than other tumours, we investigated whether this was a weight/size effect. Identified groups were rearranged and weight shown in figure S4(B). No significant differences in weight were identified between groups with weight overlaps observed between all groups (figure S4(C)). Cell type CAM xenografts were then plotted for weight and while 7 T was noticeably smaller (figure S4(D)), there was enough group/cell type weight overlap which did not correspond to the observed groupings (figure S4(A)) to suggest that weight did not influence PCA spread (figure S3(B)).

Nineteen compounds were determined to be the main drivers of group separation in the PLS-DA, figure 2(B), with FDR of *p* < 0.005 and cross validation with original data files to determine valid signal and exclude artefacts. These nineteen compounds were given tentative identification (table 1) and used in subsequent analysis.

To further support the identification of these compounds, random forest classification was applied. Mean decrease accuracy values are given in table 1 and top 15 compounds are given in figure S4, all of which are also top identifiers in the PLS-DA model, thereby supporting the PLS-DA model.

### 4.3. MPM classification with multivariate statistical analysis

**Figure 3.**
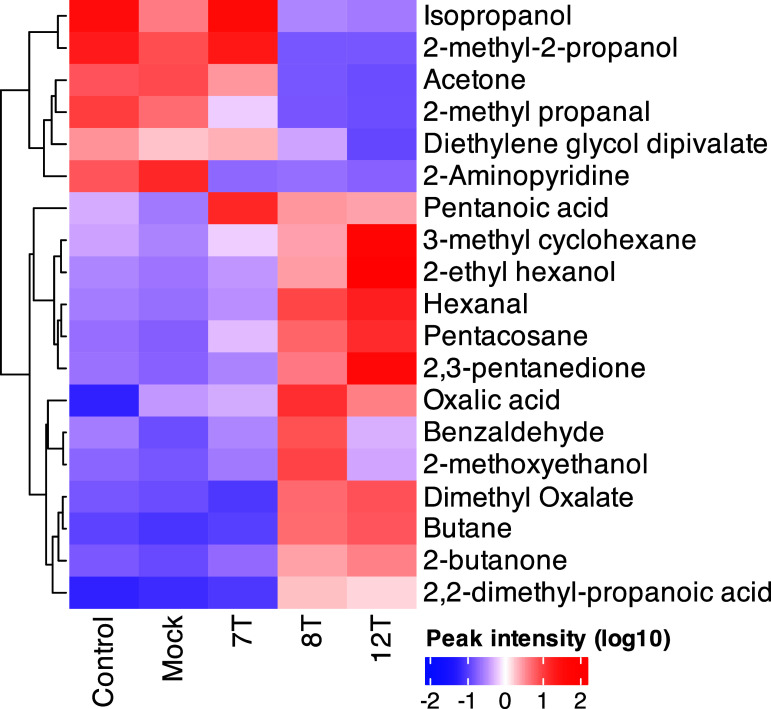
Heat-map of peak intensities (log10) for significantly altered volatile organic compounds (VOCs) between control, mock, 7 T, 8 T and 12 T CAM groups. VOCs were first determined to be significantly different from their relative RNAlater controls (Students *T*-test *p* < 0.05), these significantly different VOCs were then compared between groups using multivariate (partial least-squares discrimination analysis) and univariate analysis (Students *T*-test, *p* < 0.05).

The normalised peak intensity (log10) for the nineteen significantly altered compounds were visualised using heatmap to determine which compounds were important in describing groups. All nineteen compounds were further visualised by dot plots (figure S5). Six compounds (isopropanol, 2-methyl-2-propanol, acetone, 2-methyl propanal, diethylene glycol dipivalate and 2-amino pyridine) were increased in the control and mock CAM groups (figures 3 and S6(B), (C), (E), (G), (O), (Q)), suggesting compound consumption by tumours. 7 T CAM xenografts were similar to control and mock in the heatmap, supporting the PCA in figure 2(A). In contrast, elevated levels of pentanoic acid were seen in all three xenograft types relative to controls, and this was highest in the 7 T group (figure S5(R)). 8 T and 12 T epithelioid CAM xenografts were visually separated from other groups in the heatmap with similarly elevated levels of tert-butyl-alcohol, butane, 2-butanone and 2,2-dimethyl propanoic acid, compared to other groups (figures 3 and S6(D), (F), (H), (K), (P)). 8 T enriched compounds were primarily oxalic acid, benzaldehyde and 2-methoxyethanol (figures 3 and 6(A), (L), (M)) whereas 12 T had the highest levels of 3-methyl cyclohexane, 2-ethyl hexanol, hexanal, pentacosane and 2,3-pentadione (figures 3 and S6(I), (J), (N) and (S)).

### 4.4. Sensitivity and specificity of MPM VOC profiles

**Figure 4.**
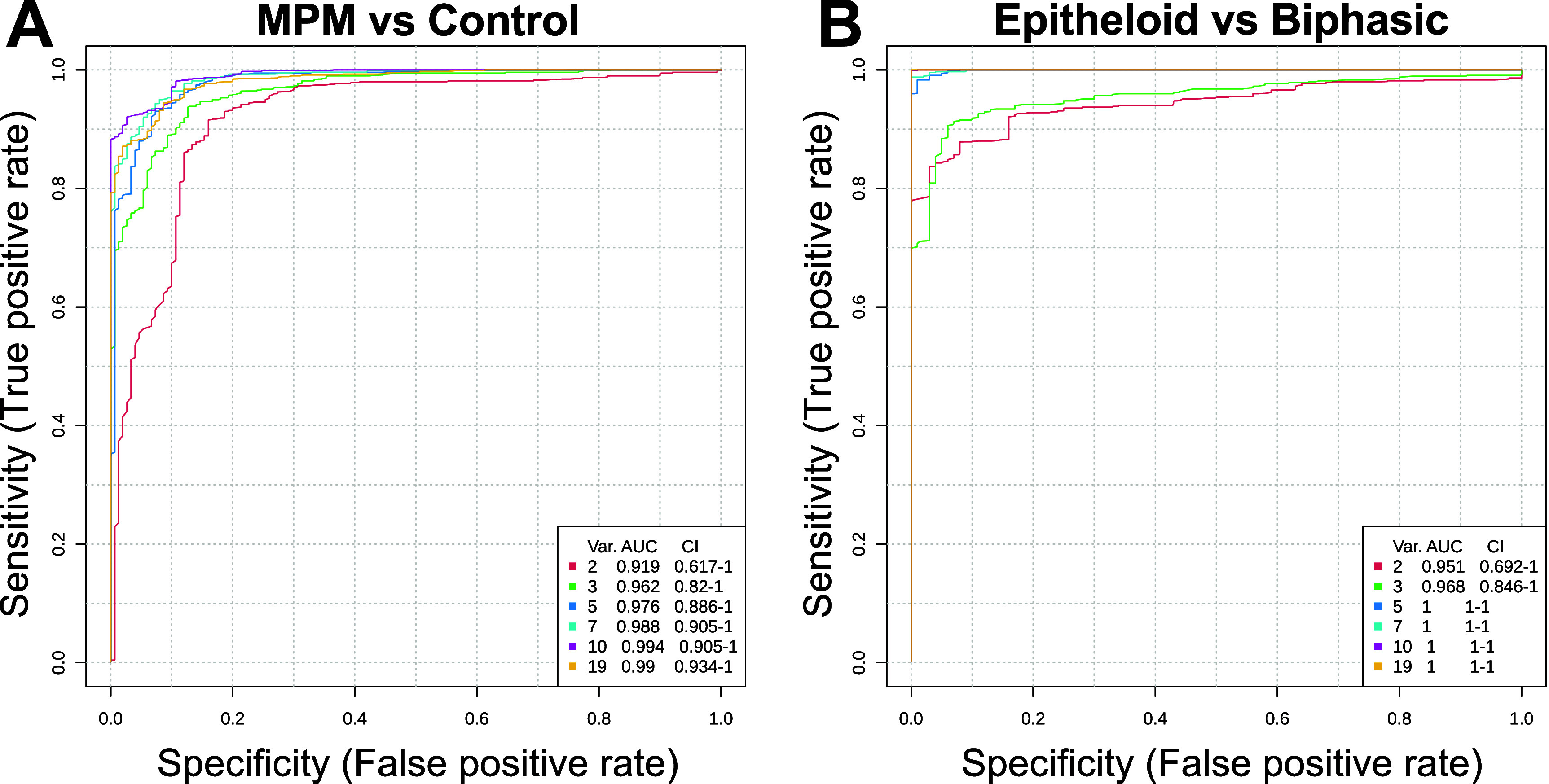
Multivariate receiver operating characteristic (ROC) curves created by MetaboAnalyst 6.0 from 5 different biomarker models considering different numbers of features (2–19). (A) MPM CAM xenograft groups (7 T, 8 T, 12 T) versus CAM controls (mock, control) and (B) epithelioid MPM (8 T & 12 T) versus biphasic MPM (7 T) CAM xenografts.

To determine the sensitivity and specificity of the MPM VOC profiles, multivariate ROC curves were generated using the normalised peak intensity (log10) for the nineteen significantly altered VOCs in Metaboanalyst (figure 3) Data for the MPM CAM xenografts (7 T, 8 T, 12 T) were grouped together and compared to the combined control CAM samples (mock and control) (figure 4). The area under the curve values when comparing MPM to Controls were close to 1 (0.919 for 2 variables and 0.99 for all nineteen), indicating strong discrimination for the presence of an MPM tumour when using this panel of VOCs (figure 4(A)). The first two variables as shown in red in figure 4(A) correspond to the top two compounds presented shown in red in figure S7, 2 aminopyridine and 2 methyl propanal. Each variable set is colour coded and labelled in figure S7 to show which compounds are included in each model.

Discrimination of epithelioid MPM cell line xenografts (8 T and 12 T) from the biphasic CAM xenograft was 0.951 using 2 variables and 1 for all variables over 5 (figure 4(B)), showing identification of MPM sub-type in this limited set of cells. Compounds used in the model are presented in figure S8.

## 5. Discussion

This work presents the first example of CAM xenografts as a model for VOC biomarker discovery. Research successfully demonstrates the capture, analysis and separation of VOC profiles for CAM xenografts from those of controls. Preliminary data suggest that this method may also be capable of distinguishing biphasic from epithelioid MPM, although the number of groups would need to be expanded to explore this further. Furthermore, nineteen VOCS are presented; oxalic acid, isopropanol, acetone, butane, 2-methyl-2-propanol, 2-methyl propanal, 2-butanone, 3-methyl cyclohexane, 2-ethyl hexanol, 2,3-pentanedione, benzaldehyde, 2-methoxyethanol, hexanal, 2,2-dimethyl-propanoic acid, 2-aminopyridine, diethylene glycol dipivalate, dimethyl oxalate, pentanoic acid, and pentacosane as key VOCs in separation of MPM sub-types and MPM xenografts from controls.

### 5.1. Alternative VOC analysis with CAM xenografts

Two-dimensional (2D) cell culture systems are limited in their ability to model pathology in biological systems, including cancer biology [24]. Research for volatile biomarker discovery, such as the work presented here, may therefore improve cell-to-breath translational biomarkers by using more complex models of disease. CAM xenografts present an opportunity to rapidly and cheaply model pathophysiology in a controlled environment, providing a complex, more biologically relevant pre-clinical model for the study of VOCs [16]. This work demonstrates the potential of CAM models for VOC biomarker discovery in MPM, and in cancer more generally.

Both PCA and PLS-DA score plots revealed separation of groups, with improved clustering in the PLS-DA score plot (figure 1), allowing separation of 7 T from controls and separation of the 8 T from the 12 T group. Considering the PCA and PLS-DA together, this supports the reliability of the data, as PLS-DA has been reported as over-optimistic within metabolomics workflows [25]. To further expand upon this, random forest classification was also applied, which also identified the same compounds as key separators between groups. The compounds presented in figure 3 are therefore compounds which distinguish experimental groups throughout these different multivariate analytical methodologies. Both score plots showed separation of the MPM xenografts from the Mock/Control groups (figure 4) in line with previous results that discriminated MPM patients from healthy controls based on breath VOC profiles [8–10]. Furthermore, we were able to show separation of the biphasic 7 T from the epithelioid 8 T and 12 T cell types (figure 2(B)). These differences are driven in part by the variations in biomarkers presented in supplementary figure 4. Variations in VOC profiles between biphasic and epithelioid MPM xenografts have also been previously shown in analysis of 2D cell cultures [12, 13]. As such, the current results appear to be in line with some of the main findings in MPM breath research, supporting the use of CAM xenograft models for VOC biomarker discovery both in MPM and for other studies.

### 5.2. MPM specific VOCs

The generation of a panel of VOC biomarkers for MPM diagnosis has the potential to increase diagnostic accuracy [5]. The CAM model can in future studies help support our understanding of the origin of VOCs through complementary metabolomic studies. Importantly, we have detected a set of compounds (table 1) which show some similarity with published breath studies for MPM patients [8–10]. While single VOC biomarkers have been shown to translate poorly between studies [5], chemical classes or compound characteristics can often be similar [5]. de Gennaro *et al* [10] reported nonanal, and aldehyde methyl cyclopentane and cyclohexane, which are cyclic alkanes, as discriminating features of MPM, whereas we reveal hexanal and cyclohexane (an aldehyde and cyclic alkane) as important features of 8 T and 12 T MPM CAM xenograft VOC profiles [5, 5, 8–10].

This is the first study to analyse VOCs from CAM samples with further development required to fully understand origins of VOCs. VOC biomarkers confer underlying features of tumour growth and pathophysiology. These compounds can originate from characteristics of tumours such as increased oxidative stress, lipid peroxidation, hypoxia, switches in metabolism and inflammation [5]. Whilst many of the mechanisms of VOC production are not yet known we have attempted to describe the potential origins of the compounds presented in figure 3 in the following paragraphs.

While it is unclear where metabolic cyclic hydrocarbons originate, hexanal is a commonly reported product of increases in lipid peroxidation and alterations in alcohol metabolism [5, 26, 27]. Increases in hexanal from 8 T and 12 T cells may be supported by the relative decreases in isopropanol and 2-methyl-2-propanol (figures S5(B) and (E)).

Isopropanol (2-propanol) has previously been reported to separate biphasic from epithelioid MPM cells [13]. 1-propanol, an analogue of isopropanol, was also previously used in a panel of VOCs to discriminate MPM patients from healthy controls [8]. 2-methyl-1-propanol was also found to be important in the discrimination of MPM patients from asbestos-exposed individuals [9]. Examples such as these could reflect the GC-MS methodology and subsequent tentative identification process that was used to identify VOCs [5]. Compounds with a very similar molecular structure can produce very similar ionisation patterns, leading to such discrepancies in identification. It is also possible that the metabolism underlying VOC production can produce compounds with a very similar structure at the same time [28].

Alterations in ketones can be signals of alterations in glucose metabolism, fatty acid and isopropanol metabolism [5, 29], furthermore, alcohols can be produced from alcohol metabolism [5, 30], suggesting the ketones and alcohols presented are metabolically linked processes. Acetone is one of the most intensively studied VOCs and has long been associated with the sweet scent that can be present on diabetic individuals’ breath [31]. 2-butanone has also been identified across several VOC studies in lung cancer [5, 32]. Increased reactive oxygen species (ROS) and oxidative stress leaves cellular macromolecules susceptible to oxidative attack, with lipid peroxidation a key driver of VOC production [33]. This VOC production process is in line with asbestos-induced carcinogenesis within MPM and pathophysiology of MPM could be a key source of VOCs, such as hypoxia [15]. The bio-persistence of inhaled asbestos fibres leads to phagocytosis and unresolved oxidative stress with high levels of ROS that, over a prolonged incubation period, lead to the development of MPM [34]. Association of VOCs with the underlying carcinogenic processes at play during MPMs incubation period could potentially provide an earlier entry point for the detection of the disease before it has reached its fatal late stages.

Oxalic acid, or oxalate, is a key compound in defining MPM from control and mock CAM in this study. It is higher in the MPM tumours and higher in the epitheloid (8 T and 12 T) cells (figure S6A). Dimethyl oxalate is also found to be higher in the MPM CAM compared to control. Interestingly, oxalate has been found to induce proliferation in breast cancer and is increased in human breast cancer tissue [35]. In lung cancer, hydroxyacid oxidase-1 (HAO1) is upregulated and results in oxalate accumulation [36]. Taken together, this may suggest that the increased oxalic acid found in MPM CAM may be linked to tumour accumulation.

Carboxylic acids and alkenes have been linked to oxidative stress and lipid peroxidation [5]. Propanoic acid has been observed in lung cancer cells [37] and it can originate from the fermentation of undigested carbohydrates by intestinal anaerobes [38]. Further research is required to elucidate the origins of the biomarkers presented in this research within the context of MPM. Although the majority of VOCs identified in this study have not been previously reported in the limited investigations of breath volatiles from MPM patients [8–10], the biomarker panels will facilitate targeted studies in patient populations.

We recently reported altered VOCs which distinguish MPM cell lines treated with asbestos, providing yet more potential VOC biomarkers of MPM [36]. In Bonsall *et al,* we found nonanone and decane, a ketone and alkane, as well as benzaldehyde may be indicative of a cellular response to asbestos exposure. Moreover, for asbestos-treated MSTO-211H, a biphasic MPM cell line, oxalic acid dibutyl ester was found to be an altered VOC. These findings support the report of variants of oxalic acid (figure 3) along with various aldehydes and aromatic compounds in this study.

Here we report VOCs in the headspace of samples of excised tumours using methods previously reported [19]. Briefly, excised tumours are placed in RNAlater placed at −80, freeze thawed and then VOCs measured in the headspace. This approach is based on VOCs which are retained in the tumour and are preserved until the point of analysis. This method provides a snapshot of VOCs present in the tumours at the point of excision and is a proxy for direct VOC metabolites. Indeed, we identified VOCs in this study which are found in the headspace of cells in culture [36] and these are importantly also reported in the breath of MPM patients [8–10], suggesting that this methodology presents a powerful approach. This particular methodology may be beneficial in the confirmation of tumour type and/or differentiation of histological subtype of tissue samples obtained during patient biopsies, where immediate headspace analysis may not be feasible or available. Freezing of samples also offers extended storage options which may be advantageous in other situations such as facilitating multicentre sampling.

## 6. Conclusions

A breath test in clinical practice will likely analyse the patterns and levels of multiple VOCs thereby increasing sensitivity and specificity compared to targeting a single compound. The correlation of VOCs across multiple malignancies may also shift the focus of a breath test from a specific cancer type towards identifying signs of malignancy in general. In terms of MPM it may also be important to target individuals that are susceptible to MPM development, such as those with a known exposure to asbestos or with asbestos related diseases. The non-invasive nature of breath test makes it suitable for monitoring at risk individuals and the early identification of VOCs associated with disease development. Large-scale patient breath studies provide correlations between specific diseases, such as MPM, and VOC profiles. In contrast, the CAM model provides a novel, biologically representative method of analysing VOCs *in vitro* that can be used to better understand these compounds in a controlled experimental environment and accelerate the field of breath research.

## Data Availability

All data that support the findings of this study are included within the article (and any supplementary files).

## Rights retention statement

For the purpose of open access, the author has applied a Creative Commons Attribution (CC BY) licence to any Author Accepted Manuscript version of this paper, arising from this submission.
